# Acquired transmissibility of sheep-passaged L-type bovine spongiform encephalopathy prion to wild-type mice

**DOI:** 10.1186/s13567-015-0211-2

**Published:** 2015-07-13

**Authors:** Hiroyuki Okada, Kentaro Masujin, Kohtaro Miyazawa, Takashi Yokoyama

**Affiliations:** National Institute of Animal Health, National Agriculture and Food Research Organization (NARO), Tsukuba, Ibaraki Japan

## Abstract

L-type bovine spongiform encephalopathy (L-BSE) is an atypical form of BSE that is transmissible to cattle and several lines of prion protein (PrP) transgenic mice, but not to wild-type mice. In this study, we examined the transmissibility of sheep-passaged L-BSE prions to wild-type mice. Disease-associated prion protein (PrP^Sc^) was detected in the brain and/or lymphoid tissues during the lifespan of mice that were asymptomatic subclinical carriers, indicating that wild-type mice were susceptible to sheep-passaged L-BSE. The morphological characteristics of the PrP^Sc^ of sheep-passaged L-BSE included florid plaques that were distributed mainly in the cerebral cortex and hippocampus of subsequent passaged mice. The PrP^Sc^ glycoform profiles of wild-type mice infected with sheep-passaged L-BSE were similar to those of the original isolate. The data indicate that sheep-passaged L-BSE has an altered host range and acquired transmissibility to wild-type mice.

## Introduction

Bovine spongiform encephalopathy (BSE) was originally thought to be caused by a single prion strain, based on analysis of its biological and biochemical characteristics. However, since 2003, different pathological and molecular phenotypes of BSE (known as atypical BSE) have been reported in approximately 90 cases worldwide, mainly in aged cattle. Currently, atypical BSE is classified into two groups depending on whether the proteinase K (PK)-resistant abnormal, disease-associated form of the prion protein (PrP^Sc^) has higher (H-BSE) or lower (L-BSE) molecular mass than that of classical (C-) BSE [1]. The origins of atypical BSE remain obscure; it is unlike C-BSE, but it is possibly a sporadic form of BSE [2].

Experimentally, L-BSE prions have shown transmissibility by intracerebral challenge to cattle [3-6]; bovinized [7-10], ovinized [7,10,11], and humanized prion protein (PrP) transgenic mice [12]; Syrian hamsters [13,14], and non-human primates [15] with a shorter incubation period than C-BSE. In contrast, L-BSE was transmitted to sheep with a longer incubation period than C-BSE [10,16]. L-BSE identified in Italy, also known as bovine amyloidotic spongiform encephalopathy (BASE), was transmissible to wild-type mice after subsequent passages, with an altered C-BSE-like phenotype [17]. In this study, we examine the biological and biochemical characteristics of ARQ/ARQ sheep-passaged L-BSE (L-BSE/sheep) to evaluate any alteration or consistency in the biological phenotypes during inter-species transmission.

## Materials and methods

### Experimental design

All the experiments involving animals were performed with the approval of the Animal Ethics Committee and the Animal Care and Use Committee of the National Institute of Animal Health (approval ID: 10–005 and 11–008). Fifteen 3-week-old outbred ICR (CD-1) mice (Japan SLC Inc., Shizuoka, Japan) were inoculated intracerebrally with 20 μL of 10% brain homogenates of Japanese L-BSE [18] passaged in an ARQ/ARQ Cheviot ewe [16]. Inoculated mice were maintained in an animal biological containment level 3 facility under identical environmental conditions (21–22 °C, 50–60% relative humidity) and examined daily for neurological signs of the disease.

### Histopathology and immunohistochemistry

At necropsy, the left hemisphere and selected tissues including the lymphoid organs were removed and fixed in 10% buffered formalin containing 10% methanol. Formalin-fixed tissues were immersed in 98% formic acid for 60 min to reduce the infectivity, embedded in paraffin, and sectioned for histological evaluation by staining with hematoxylin and eosin (HE), and using PrP^Sc^ immunohistochemistry (IHC). Selected sections were stained with phenol Congo red and examined under a polarizing microscope, and the presence of amyloid was confirmed by observation of its characteristic dichroism [19].

### PrP^Sc^ immunohistochemistry

After appropriate epitope retrieval with either hydrate autoclaving or a combination of enzymatic and chemical treatment, IHC was carried out using the monoclonal antibodies (mAbs) 2G11, 12F10, or SAF84 (SPI-Bio, Montigny le Bretonneux, France) followed by an anti-mouse, universal horseradish peroxidase (HRP)-conjugated polymer (Nichirei Histofine Simple Stain MAX-PO (M); Nichirei Biosciences Inc., Tokyo, Japan) as the secondary antibody, and visualized with 3,3′-diaminobenzedine tetrachloride as the chromogen, as previously described [20]. Finally, the sections were lightly counterstained with Mayer’s hematoxylin.

### Western Blotting (WB)

The right hemisphere and spleen were removed and stored at −80 °C until use. The tissues (200 ± 10 mg) were homogenized at 20% concentration (w/v) in a buffer containing 100 mM NaCl and 50 mM Tris–HCl (pH 7.6). The homogenates (250 μL) were mixed with an equal volume of detergent buffer containing 4% (w/v) Zwittergent 3–14 (EMD Millipore, Billerica, MA, USA), 1% (w/v) Sarkosyl, 100 mM NaCl, and 50 mM Tris–HCl (pH 7.6), and treated with 6.25 μL of 40 mg/mL collagenase. The sample was then digested with 40 μg/mL proteinase K (PK; Roche Diagnostics, Basel, Switzerland) and the digestion was terminated using 2 mM 4-(2-aminoethyl) benzenesulfonyl fluoride hydrochloride (Pefabloc; Roche Diagnostics). After PK treatment, the samples were mixed with a 2-butanol: methanol mixture (5:1) and centrifuged at 20000 × *g* for 10 min. The pellets were mixed with a gel-loading buffer containing 2% sodium dodecyl sulfate, boiled for 5 min before electrophoresis, and loaded onto a 12% polyacrylamide gel. The separated proteins were transferred onto an Immobilon-P polyvinylidene fluoride membrane (EMD Millipore). The blotted membranes were incubated with the mAbs SAF84 and T2 [21] followed by incubation with HRP-conjugated anti-mouse IgG (Jackson ImmunoResearch, West Grove, PA, USA). Signals were developed using a chemiluminescent substrate (SuperSignal; Pierce Biotechnology, Rockford, IL, USA). Blots were imaged with a FluorChem system (Alpha Innotech, San Leandro, CA, USA) and analyzed using ImageReader software (AlphaEaseFC; Alpha Innotech) after background subtraction.

To compare the molecular features of PrP^Sc^ in wild-type mice inoculated with L-BSE/sheep, the brains of wild-type mice [22,23], cattle [4,9], or sheep [16] inoculated intracerebrally with C-BSE or L-BSE from cattle (L-BSE/cattle), were also examined.

### Characterization of monoclonal antibodies

The mAbs 2G11, SAF84, and T2 were found to react with bovine, ovine, and mouse PrP; the mAb 12F10 reacts with both bovine and ovine, but not mouse PrP.

### Back passage of L-BSE/sheep in wild-type to bovinized PrP (TgBoPrP) mice

To compare the phenotypic features of L-BSE inoculum before and after passage in sheep, reverse transmission to bovinized PrP expressing transgenic (TgBoPrP) mice was carried out to examine whether the L-BSE/sheep would maintain its specific strain properties in bovids. TgBoPrP mice were kindly provided by Dr Prusiner [24]. The susceptibility of TgBoPrP mice to L-BSE has been confirmed previously [21].

### Statistical analysis

Incubation periods expressed as mean ± standard deviation of the mean (SD) and signal intensities of PK-resistant PrP^Sc^ bands were analyzed using Instat3 software (GraphPad Software; La Jolla, CA, USA) and ImageReader software (AlphaEaseFC; Alpha Innotech) after background subtraction, respectively; *p* values <0.05 were considered statistically significant.

## Results

### First and second passage of L-BSE/sheep to wild-type mice

**Figure 1:**
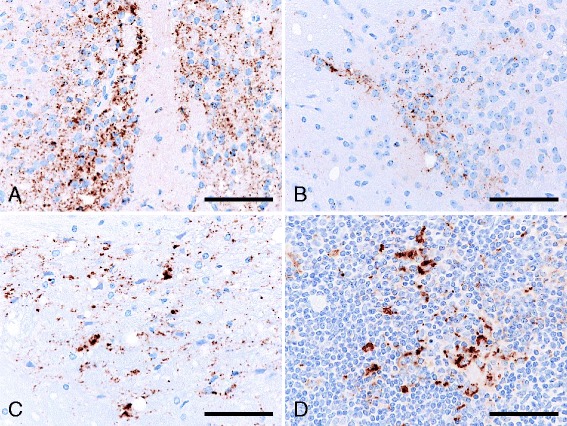
**Immunolabeled PrP** ^**Sc**^ **accumulation in the brain and lymph node of ICR mice infected with sheep-passaged L-BSE, at first passage.** PrP^Sc^ accumulated in the brain of the medial preoptic nucleus (**A**), habenular nucleus (**B**), and midbrain tegmentum (**C**), and in the follicular dendritic cells within the secondary follicle of renal lymph node (**D**) of a mouse killed at 710 days post-inoculation. Immunohistochemical labeling with mAb SAF84 and hematoxylin counterstain. Scale bar = 25 μm.

The first-passaged mice with L-BSE/sheep showed no clinical signs of the disease and were sacrificed at the end of their lives, or at an earlier stage of deterioration in accordance with welfare concerns relating to animal experiments, during the period between 172 and 1012 days post-inoculation (dpi) (Table 1). A PrP^Sc^ signal was detected by WB, IHC, or both techniques in the brain from 1 case at 710 dpi, and in lymphoid tissues (including spleen, lymph nodes, tonsils, and Peyer’s patches) from 9 of 15 mice after 200 dpi after the first passage. Positive IHC results in a mouse were typically composed of sparse granular deposits in some areas of the brain such as the vestibular nucleus or dorsal motor nucleus of the vagal nerve, midbrain tegmentum, hypothalamus, medial preoptic nucleus, and habenular nucleus (Figure 1). In addition, intense PrP^Sc^ immunolabeling in the follicles of lymphoid tissues was confined to follicular dendritic cells (Figure 1D). The mAbs 2G11 and SAF84 produced positive results in the sections, but mAb 12F10 was negative. However, transmission of the original isolate of L-BSE/cattle into wild-type mice was inefficient, and resulted in no clinical signs of the disease at the end of the lifespan, and an absence of positive signals in the brains and lymphoid tissues by WB and IHC analyses (Table 1).

**Table 1:** Transmission of L-BSE isolates to mice^a^

Hosts	Source	Inoculum	Passage	Survival periods (days)^b^	Range (days)	Proportion of PrP^Sc^ mice^c^	
						Brain	Spleen
ICR [14]	L-BSE/cattle [18]	Brain	1	651 ± 153	322–953	0/35	0/35
ICR	L-BSE/cattle passaged in ICR mice	Brain	2	666 ± 198	386–937	0/10	0/10
C57BL/6^d^	L-BSE/cattle [18]	Brain	1	713 ± 42	673–757	0/5	0/5
ICR	L-BSE/sheep [16]	Brain	1	569 ± 262	172–1012	1/15	9/15
ICR	L-BSE/sheep passaged in ICR mice	Spleen	2	691 ± 61	639–765	6/6	6/6
TgBoPrP	L-BSE/cattle [18]	Brain	1	195 ± 6	187-211	18/18	0/18
TgBoPrP	L-BSE/cattle passaged in TgBoPrP mice	Brain	2	152 ± 2	148-155	24/24	0/24
TgBoPrP	L-BSE/sheep [16]	Brain	1	249 ± 28	234-298	5/5	0/5
TgBoPrP	L-BSE/sheep passaged in TgBoPrP mice	Brain	2	269 ± 17	248-305	13/13	0/13

^a^TgBoPrP, bovine PrP expressing transgenic; L-BSE, L-type bovine spongiform encephalopathy; PrP^Sc^, disease-associated prion protein.

^b^mean ± standard deviation.

^c^Number of PrP^Sc^ positive tissue samples per number of examined tissue samples. Results from either western blot, immunohistochemistry, or both.

^d^Unpublished data from our laboratory.

**Figure 2:**
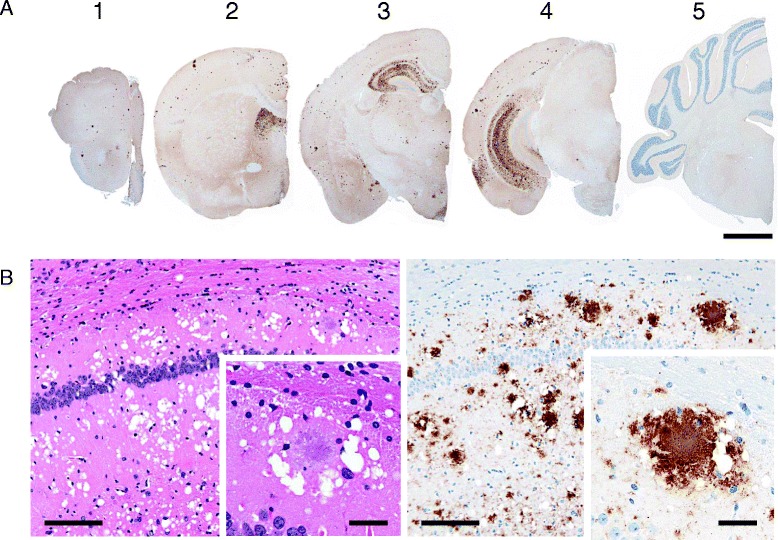
**Neuroanatomical distribution and morphological patterns of PrP** ^**Sc**^ **accumulation in the brain of ICR mice infected with sheep-passaged L-BSE, at second passage.** **A** The 5 coronal brain sections are as follows: 1. frontal cortex, 2. septal level, 3. hippocampus and thalamic level, 4. midbrain, and 5. medulla with cerebellum. PrP-plaque deposits are abundant throughout the cerebral gray matter (1 to 4) and hippocampus (3 and 4). Granular and aggregated PrP^Sc^ accumulated in the septal nuclei (2), hypothalamus (3), and temporal cortex (4). Scale bar = 1 mm. **B** Histopathology and PrP^Sc^ immunohistochemical analysis with mAb SAF84 revealed plentiful florid plaques accompanied by granular and aggregated PrP^Sc^ deposits in the hippocampus. Insets show high-magnification images of a florid plaque. Scale bar = 100 μm, scale bar in inset = 25 μm.

The inoculum prepared from PrP^Sc^-positive spleens of mice was used for subsequent second passages in ICR mice (Table 1). The long survival period of 691 ± 61 days (*n* = 6) was statistically unchanged on second passage, without any clinical signs and a 100% transmission rate. Histopathological examination showed prominent florid and non-florid plaques with large confluent vacuoles in the cerebral cortex and hippocampus of all inoculated mice (Figure 2). Florid plaques with a pale central core were generally stained pale basophilic or amphophilic with HE and positively with Congo red under polarized light. A few non-florid plaques were also detected in the thalamus, midbrain tegmentum, and vestibular nucleus. In addition, coarse granular PrP^Sc^ and PrP^Sc^ aggregates accumulated in the septal nuclei, diagonal band of Broca, hippocampus, hypothalamus, and several limited regions of the cerebral cortex, the olfactory bulb, and the olfactory tract of the frontal cortex (Figure 2B). However, some variation in the intensity of immunolabeling was observed throughout the brain. In addition to the brains of these mice, positive PrP^Sc^ immunolabeling was detected in the retina, but not in the spinal cords, trigeminal and dorsal root ganglia, and muscle bundles of skeletal muscle fibers. The pathological results revealed striking differences between first- and second-passaged mice; however the findings from the immunolabeling reactions conducted using the mAbs SAF84, 2G11, and 12F10 for the second-passaged mice were similar to those for the first-passaged mice.

### Molecular features of PK-resistant PrP^Sc^ in wild-type mice affected with L-BSE/sheep

**Figure 3:**
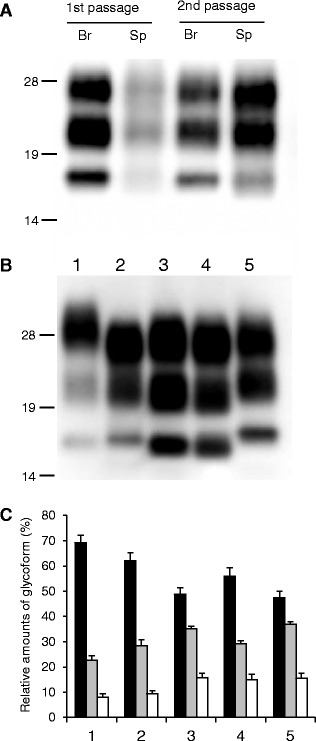
**Western blot analysis of proteinase-K resistant PrP** ^**Sc**^ **analyzed using monoclonal antibody T2.** **A** PrP^Sc^ in the brain (Br) and spleen (Sp) of wild-type mice inoculated with sheep-passaged L-BSE at the first and second passage. All samples were digested with 50 μg/mL of proteinase-K at 37 °C for 1 h. Lanes from left to right were loaded with 0.625, 5, 0.0125, and 0.36 mg tissue equivalent, respectively. The molecular markers are shown on the left (kDa). **B** PrP^Sc^ in the brain of C-BSE- and L-BSE-affected cattle and mice. Lane 1: C-BSE affected cattle, Lane 2: C-BSE affected ICR mouse, Lane 3: L-BSE affected cattle, Lane 4: L-BSE affected sheep, and Lane 5: sheep-passaged L-BSE affected ICR mouse at second passage. Lanes 1, 3, and 4, and Lanes 2 and 5 were loaded with 1.25 and 0.125 mg tissue equivalent, respectively. **C** Quantification of the relative amounts of the di-, mono-, and unglycosylated forms of PrP^Sc^ from the brain. The column numbers are as listed in (**B**). Bar diagram indicates the diglycosylated form (black), monoglycosylated form (gray), and unglycosylated form (white). Data are expressed as mean ± standard deviation of triplicate experiments.

Conventional WB features of PK-resistant PrP^Sc^, such as the electrophoretic mobility and the relative proportions and pattern of the glycoforms obtained using the mAbs T2 and SAF84, were similar between the first and the second passage in the brain and spleen of wild-type mice inoculated with L-BSE/sheep (Figure 3A). The molecular mass of the unglycosylated form was ~18 kDa in cattle, sheep, and wild-type mice affected with L-BSE. Interestingly, the three bands in samples from wild-type mice represented higher molecular mass forms compared with those of cattle and sheep affected with L-BSE (Figure 3B). Of note, the PrP^Sc^ glycoprofile of L-BSE/sheep-affected wild-type mice resembled those of L-BSE-affected cattle and sheep, but was distinct from those of C-BSE-affected cattle and wild-type mice (Figure 3C).

### Reverse transmission of L-BSE/sheep to TgBoPrP mice

**Figure 4:**
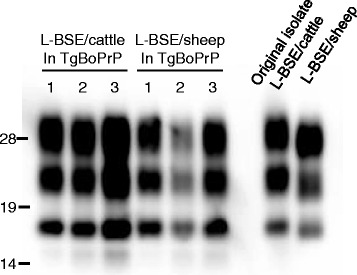
**Western blot analysis of proteinase-K resistant L-BSE PrP** ^**Sc**^ **in TgBoPrP mice before and after passage in sheep.** The brain samples of TgBoPrP mice inoculated intracerebrally with either L-BSE/cattle or L-BSE/sheep were analyzed. PrP^Sc^ in the twice-passaged mice was detected with mAb T2. The numbers indicate three different mice.

Transmission of L-BSE/cattle to TgBoPrP mice resulted in a mean incubation period of 195 ± 6 (*n* = 18) and 152 ± 2 days (*n* = 24) at primary and secondary passage, respectively. In contrast, the mean incubation period of L-BSE/sheep to TgBoPrP mice was 249 ± 28 (*n* = 5) and 269 ± 17 days (*n* = 13) at primary and secondary passage, respectively, which indicated a significantly longer incubation period compared to L-BSE/cattle, the original isolate (Table 1). However, pathological changes including lesion profiles and distribution patterns or types of PrP^Sc^, and molecular features of PK-resistant PrP^Sc^ in the brains of TgBoPrP mice inoculated with L-BSE/sheep were identical to those in mice inoculated with L-BSE/cattle (Figure 4). In addition, PK-resistant PrP^Sc^ in TgBoPrP mice inoculated with either L-BSE/sheep or L-BSE/cattle showed similar biochemical profiles to L-BSE/cattle, the original isolate.

### No transmissibility of L-BSE/cattle to wild-type mice

Wild-type mice inoculated with L-BSE/cattle did not develop prion diseases. In addition, a second “blind” passage transmission of brain homogenates from L-BSE/cattle-inoculated ICR mice to normal ICR mice (*n* = 10) showed a lack of any evidence of transmission during their lifespan by both WB and IHC tests (Table 1).

## Discussion

A transmission study performed on experimental animals is a useful approach for the isolation and characterization of prion strains. In the present study, we were able to demonstrate the transmissibility of L-BSE/sheep to wild-type mice across a species barrier during their lifespans, in the absence of clinical signs of the disease. According to the protein-only hypothesis, PrP^Sc^ is the principal component of the infectious agent and the conformational differences in PrP^Sc^ determine the strain phenotype [25]. The PrP^Sc^ conformational change at the molecular level is believed to be essential during interspecies transmission. The conformational transition of mouse PrP^Sc^ might occur during adaptation in the sheep-to-wild-type mice transmission of L-BSE prions [26]. Since this mechanism would be limited to developing heterologous PrP^Sc^ aggregation before the mice reach the end of their lifespan at first passage, mouse PrP^Sc^ would fit the given environment and propagate in mouse brains with a 100% attack rate of the infection at second passage [27]. Interestingly, PrP^Sc^ propagated substantially in the lymphoid tissues of more than half of the affected mice, and was detected there as early as 200 dpi. This was not the case in the brain, except for only 1 case before 710 dpi, suggesting that L-BSE/sheep showed apparent lymphotropism in wild-type mice at an early stage of infection, from the first passage. Propagation of PrP^Sc^ in lymphoid tissues has been reported in ovine PrP transgenic mice carrying the ARQ allele (TgOvPrP) that are affected by L-BSE/sheep [10], whereas lymphoid PrP^Sc^ accumulation has not been detected in cattle [5,28] or sheep [16] affected by L-BSE and wild-type mice affected with C-BSE [29]. In addition, the presence of clear lymphotropism in animals with a subclinical status has been reported in transgenic mouse models affected by BSE, chronic wasting disease, or Sc237 [30]. Thus, some prion strains exhibit a much greater preference for propagating in lymphoid tissue than in nervous tissue, and at an early stage of infection via cross-species transmission, notwithstanding intracerebral exposure [30,31]. Consequently, the accumulation of PrP^Sc^ in lymphoid tissues does not necessarily result in neuroinvasion [31]. In the experiment involving secondary transmission using spleen homogenates from primary-passaged mice, the disease was found to progress to the subclinical stage of infection only after 600 days, although conspicuous PrP^Sc^ deposits accumulated in the brain. This result indicates that L-BSE/sheep may not have pathogenicity and virulence towards wild-type mice. The discrimination and typing of prion strains are dependent on biological characteristics that include clinical signs, incubation times, histopathological vacuolar lesion profiles, PrP^Sc^ deposition patterns in the brain, and the biochemical features of PrP^Sc^ over several mouse passages [32,33].

In the results of WB tests, a strain-specific molecular signature such as the glycoform pattern was conserved in the transmitted wild-type mice. The occurrence of size shifts in PK-digested PrP^Sc^ has been reported in cross-species transmission of sporadic Creutzfeldt-Jakob disease (CJD) to humanized transgenic mice [34], variant CJD to wild-type mice [35], and hamster Sc237 to wild-type mice [36]. The transmission of L-BSE/sheep did not alter the glycoprofile of PrP^Sc^, but gained the transmissibility to wild-type mice. Although the key event that determines the shift in the size of PK-resistant PrP^Sc^ remains unknown, it seems likely that the molecular characteristics may be influenced by the host-environment factors rather than the nature of the prion strain. The specific strain features of L-BSE observed in TgBoPrP mice affected with L-BSE/cattle [22,37] or L-BSE/sheep were consistent after the passage transmission in sheep.

To the best of our knowledge, the transmission of L-BSE/cattle to wild-type mice has only been reported in one study, and even in this case the L-BSE prions were converted to a C-BSE-like prion using serial passages, and had indistinguishable phenotypic traits compared with mouse-passaged C-BSE [17]. A phenotypic change during the transmission of prions is a common phenomenon across a species barrier [33,38,39]. However, the reasons for the discrepancy between this study here and another, suggesting that BASE prion converts into C-BSE-like phenotypes during interspecies transmission in wild-type mice [17] are unknown. Several possible reasons are concisely considered: (1) cross contamination may occur during the inoculation procedure, (2) undetectable levels of C-BSE agent by WB analysis emerge in the brain of mice challenged with BASE at the first passage, thereafter inoculated mice develop the disease in subsequent passages [29], (3) L-BSE could generate at least 2 types of prions in wild-type mice: one showing L-BSE phenotypic properties and the other producing C-BSE-like signatures, (4) differences of unidentified prion-related host factors between outbred (ICR) and inbred (C57BL/6, SJL, or RIII) mice may have influenced the emergence of C-BSE-like prions during the cross-species transmission, and (5) differences of experimental procedures including prepared inocula and/or challenge routes of the infection may have influenced the propagation and/or generation of PrP^Sc^ in the brain. The first two possibilities were completely ruled out by the authors [17]. The last possibility, is that mice were inoculated by a combination of intracerebral and intraperitoneal routes with a thalamic sample at first passage and with brain pools prepared from C57Bl/6 or SJL mice at second passage [17], should help address this issue [40]. No transmissibility including lymphotropism was found on the first passage in the Italian study. Although PrP^Sc^ was undetectable in the brain of these mice, a faint positive signal was identified in one RIII mouse that showed biochemical characteristics of PrP^Sc^ identical to those of C-BSE-infected mice by WB analyses.

Four L-BSE isolates from Japan [18], Germany [8], France [41], and Canada [42] were transmitted to TgBoPrP mice and no distinctive differences were detected in their pathological and molecular signatures [37]. These results suggest that the Japanese L-BSE isolates (BSE/JP24) used in this study may be identical to those from Canadian and European L-BSE cases examined. However, further studies regarding transmission to wild-type mice using these L-BSE isolates is now under consideration to address the issue that L-BSE prions from cattle are not transmitted to wild-type mice, which is a general phenomenon for L-BSE prions that is not restricted to the isolate used in this study. Furthermore, reverse transmission of L-BSE/sheep to TgBoPrP mice showed that L-BSE prions retained their pathological and biochemical signatures after passage in ARQ/ARQ sheep, which was in accordance with the findings of a previous study [10]. However, the mean incubation period of L-BSE/sheep affected TgBoPrP mice was much longer than that of L-BSE/cattle. Although the exact reason for the discrepancy that determines the incubation periods remains unknown, the environment in sheep as an intermediate host may influence the incubation periods.

Finally, the results indicate that L-BSE/sheep is transmissible to wild-type mice and it results in low virulence compared with C-BSE [23]. In contrast, experimental transmission of sheep-passaged C-BSE to bovine PrP transgenic mice induced a shorter incubation period and more severe neuropathological changes compared to cattle C-BSE, suggesting that the pathogenic properties of the C-BSE agent were altered during the inter-species transmission, making it more virulent in sheep [43]. Amino acid differences between the host PrP^C^ and the PrP^Sc^ of inocula result in species barriers to the cross-species transmission of prions [33]. In this context, the transmission of L-BSE to wild-type mice may be influenced by the ovine PrP amino-acid sequence. The biochemical nature of the protein in terms of its glycoform profile is identical in original L-BSE, L-BSE/sheep, and inoculated mice, even after the subsequent passage. Here, we have generated mouse-passaged L-BSE prions, which have the similar biochemical characteristics as the original cattle L-BSE. Therefore, this wild-type mouse model may be a useful experimental tool for elucidating BSE prion strains. The transmission experiment reported here shows that the host range of L-BSE prions can be extended by inter-species transmission. Further experimental transmission of L-BSE/sheep along with L-BSE into humanized PrP mice will be necessary to evaluate the potential risk for humans.

## References

[1] Jacobs JG, Langeveld JP, Biacabe AG, Acutis PL, Polak MP, Gavier-Widen D, Buschmann A, Caramelli M, Casalone C, Mazza M, Groschup M, Erkens JH, Davidse A, van Zijderveld FG, Baron T (2007). Molecular discrimination of atypical bovine spongiform encephalopathy strains from a geographical region spanning a wide area in Europe. J Clin Microbiol.

[2] Brown P, McShane LM, Zanusso G, Detwile L (2006). On the question of sporadic or atypical bovine spongiform encephalopathy and Creutzfeldt-Jakob disease. Emerg Infect Dis.

[3] Balkema-Buschmann A, Ziegler U, McIntyre L, Keller M, Hoffmann C, Rogers R, Hills B, Groschup MH (2011). Experimental challenge of cattle with German atypical bovine spongiform encephalopathy (BSE) isolates. J Toxicol Environ Health A.

[4] Fukuda S, Iwamaru Y, Imamura M, Masujin K, Shimizu Y, Matsuura Y, Shu Y, Kurachi M, Kasai K, Murayama Y, Onoe S, Hagiwara K, Sata T, Mohri S, Yokoyama T, Okada H (2009). Intraspecies transmission of L-type-like bovine spongiform encephalopathy detected in Japan. Microbiol Immunol.

[5] Konold T, Bone GE, Clifford D, Chaplin MJ, Cawthraw S, Stack MJ, Simmons MM (2012). Experimental H-type and L-type bovine spongiform encephalopathy in cattle: observation of two clinical syndromes and diagnostic challenges. BMC Vet Res.

[6] Lombardi G, Casalone C, D’ Angelo A, Gelmetti D, Torcoli G, Barbieri I, Corona C, Fasoli E, Farinazzo A, Fiorini M, Gelati M, Iulini B, Tagliavini F, Ferrari S, Caramelli M, Monaco S, Capucci L, Zanusso G (2008). Intraspecies transmission of BASE induces clinical dullness and amyotrophic changes. PLoS Pathog.

[7] Béringue V, Andréoletti O, Le Dur A, Essalmani R, Vilotte JL, Lacroux C, Reine F, Herzog L, Biacabé AG, Baron T, Caramelli M, Casalone C, Laude H (2007). A bovine prion acquires an epidemic bovine spongiform encephalopathy strain-like phenotype on interspecies transmission. J Neurosci.

[8] Buschmann A, Gretzschel A, Biacabe AG, Schiebel K, Corona C, Hoffmann C, Eiden M, Baron T, Casalone C, Groschup MH (2006). Atypical BSE in Germany–proof of transmissibility and biochemical characterization. Vet Microbiol.

[9] Fukuda S, Onoe S, Nikaido S, Fujii K, Kageyama S, Iwamaru Y, Imamura M, Masujin K, Matsuura Y, Shimizu Y, Kasai K, Yoshioka M, Murayama Y, Mohri S, Yokoyama T, Okada H (2012). Neuroanatomical distribution of disease-associated prion protein in experimental bovine spongiform encephalopathy in cattle after intracerebral inoculation. Jpn J Infect Dis.

[10] Nicot S, Bencsik A, Migliore S, Canal D, Leboidre M, Agrimi U, Nonno R, Baron T (2014). L-type bovine spongiform encephalopathy in genetically susceptible and resistant sheep: changes in prion strain or phenotypic plasticity of the disease-associated prion protein?. J Infect Dis.

[11] Baron T, Bencsik A, Biacabe AG, Morignat E, Bessen RA (2007). Phenotypic similarity of transmissible mink encephalopathy in cattle and L-type bovine spongiform encephalopathy in a mouse model. Emerg Infect Dis.

[12] Kong Q, Zheng M, Casalone C, Qing L, Huang S, Chakraborty B, Wang P, Chen F, Cali I, Corona C, Martucci F, Iulini B, Acutis P, Wang L, Liang J, Wang M, Li X, Monaco S, Zanusso G, Zou WQ, Caramelli M, Gambetti P (2008). Evaluation of the human transmission risk of an atypical bovine spongiform encephalopathy prion strain. J Virol.

[13] Nicot S, Baron T (2011). Strain-specific barriers against bovine prions in hamsters. J Virol.

[14] Shu Y, Masujin K, Okada H, Iwamaru Y, Imamura M, Matsuura Y, Mohri S, Yokoyama T (2011). Characterization of Syrian hamster adapted prions derived from L-type and C-type bovine spongiform encephalopathies. Prion.

[15] Ono F, Tase N, Kurosawa A, Hiyaoka A, Ohyama A, Tezuka Y, Wada N, Sato Y, Tobiume M, Hagiwara K, Yamakawa Y, Terao K, Sata T (2011). Atypical L-type bovine spongiform encephalopathy (L-BSE) transmission to cynomolgus macaques, a non-human primate. Jpn J Infect Dis.

[16] Matsuura Y, Iwamaru Y, Masujin K, Imamura M, Mohri S, Yokoyama T, Okada H (2013). Distribution of abnormal prion protein in a sheep affected with L-type bovine spongiform encephalopathy. J Comp Pathol.

[17] Capobianco R, Casalone C, Suardi S, Mangieri M, Miccolo C, Limido L, Catania M, Rossi G, Di Fede G, Giaccone G, Bruzzone MG, Minati L, Corona C, Acutis P, Gelmetti D, Lombardi G, Groschup MH, Buschmann A, Zanusso G, Monaco S, Caramelli M, Tagliavini F (2007) Conversion of the BASE prion strain into the BSE strain: the origin of BSE? PLoS Pathog 3:e31 10.1371/journal.ppat.0030031 PMC1817656 17352534

[18] Hagiwara K, Yamakawa Y, Sato Y, Nakamura Y, Tobiume M, Shinagawa M, Sata T (2007). Accumulation of mono-glycosylated form-rich, plaque-forming PrPSc in the second atypical bovine spongiform encephalopathy case in Japan. Jpn J Infect Dis.

[19] Sai S, Hayama M, Hotchi M (1986). A new amyloid stain by phenol Congo red. Pathol Clin Med.

[20] Okada H, Sato Y, Sata T, Sakurai M, Endo J, Yokoyama T, Mohri S (2011). Antigen retrieval using sodium hydroxide for prion immunohistochemistry in bovine spongiform encephalopathy and scrapie. J Comp Pathol.

[21] Shimizu Y, Kaku-Ushiki Y, Iwamaru Y, Muramoto T, Kitamoto T, Yokoyama T, Mohri S, Tagawa Y (2010). A novel anti-prion protein monoclonal antibody and its single-chain fragment variable derivative with ability to inhibit abnormal prion protein accumulation in cultured cells. Microbiol Immunol.

[22] Masujin K, Shu Y, Yamakawa Y, Hagiwara K, Sata T, Matsuura Y, Iwamaru Y, Imamura M, Okada H, Mohri S, Yokoyama T (2008). Biological and biochemical characterization of L-type-like bovine spongiform encephalopathy (BSE) detected in Japanese black beef cattle. Prion.

[23] Yokoyama T, Masujin K, Iwamaru Y, Imamura M, Mohri S (2009). Alteration of the biological and biochemical characteristics of bovine spongiform encephalopathy prions during interspecies transmission in transgenic mice models. J Gen Virol.

[24] Scott MR, Safar J, Telling G, Nguyen O, Groth D, Torchia M, Koehler R, Tremblay P, Walther D, Cohen FE, DeArmond SJ, Prusiner SB (1997). Identification of a prion protein epitope modulating transmission of bovine spongiform encephalopathy prions to transgenic mice. Proc Natl Acad Sci U S A.

[25] Telling GC, Parchi P, DeArmond SJ, Cortelli P, Montagna P, Gabizon R, Mastrianni J, Lugaresi E, Gambetti P, Prusiner SB (1996). Evidence for the conformation of the pathologic isoform of the prion protein enciphering and propagating prion diversity. Science.

[26] Ushiki-Kaku Y, Endo R, Iwamaru Y, Shimizu Y, Imamura M, Masujin K, Yamamoto T, Hattori S, Itohara S, Irie S, Yokoyama T (2010). Tracing conformational transition of abnormal prion proteins during interspecies transmission by using novel antibodies. J Biol Chem.

[27] Castilla J, Gutierrez-Adan A, Brun A, Doyle D, Pintado B, Ramirez MA, Salguero FJ, Parra B, Segundo FD, Sanchez-Vizcaino JM, Rogers M, Torres JM (2004). Subclinical bovine spongiform encephalopathy infection in transgenic mice expressing porcine prion protein. J Neurosci.

[28] Iwamaru Y, Imamura M, Matsuura Y, Masujin K, Shimizu Y, Shu Y, Kurachi M, Kasai K, Murayama Y, Fukuda S, Onoe S, Hagiwara K, Yamakawa Y, Sata T, Mohri S, Okada H, Yokoyama T (2010). Accumulation of L-type bovine prions in peripheral nerve tissues. Emerg Infect Dis.

[29] Lasmézas CI, Deslys JP, Robain O, Jaegly A, Beringue V, Peyrin JM, Fournier JG, Hauw JJ, Rossier J, Dormont D (1997). Transmission of the BSE agent to mice in the absence of detectable abnormal prion protein. Science.

[30] Béringue V, Herzog L, Jaumain E, Reine F, Sibille P, Le Dur A, Vilotte JL, Laude H (2012). Facilitated cross-species transmission of prions in extraneural tissue. Science.

[31] Béringue V, Le Dur A, Tixador P, Reine F, Lepourry L, Perret-Liaudet A, Haik S, Vilotte JL, Fontes M, Laude H (2008) Prominent and persistent extraneural infection in human PrP transgenic mice infected with variant CJD. PLoS One 3:e1419 10.1371/journal.pone.0001419 PMC2171367 18183299

[32] Lloyd SE, Linehan JM, Desbruslais M, Joiner S, Buckell J, Brandner S, Wadsworth JD, Collinge J (2004). Characterization of two distinct prion strains derived from bovine spongiform encephalopathy transmissions to inbred mice. J Gen Virol.

[33] Collinge J, Clarke AR (2007). A general model of prion strains and their pathogenicity. Science.

[34] Kobayashi A, Asano M, Mohri S, Kitamoto T (2007). Cross-sequence transmission of sporadic Creutzfeldt-Jakob disease creates a new prion strain. J Biol Chem.

[35] Hill AF, Desbruslais M, Joiner S, Sidle KC, Gowland I, Collinge J, Doey LJ, Lantos P (1997). The same prion strain causes vCJD and BSE. Nature.

[36] Hill AF, Joiner S, Linehan J, Desbruslais M, Lantos PL, Collinge J (2000). Species-barrier-independent prion replication in apparently resistant species. Proc Natl Acad Sci U S A.

[37] Masujin K, Miwa R, Okada H, Mohri S, Yokoyama T (2012). Comparative analysis of Japanese and foreign L-type BSE prions. Prion.

[38] Béringue V, Vilotte JL, Laude H (2008). Prion agent diversity and species barrier. Vet Res.

[39] Collinge J (2010). Prion strain mutation and selection. Science.

[40] Baron T, Vulin J, Biacabe AG, Lakhdar L, Verchere J, Torres JM, Bencsik A (2011) Emergence of classical bse strain properties during serial passages of H-BSE in wild-type mice. PLoS One 6:e15839 10.1371/journal.pone.0015839 PMC3021503 21264286

[41] Biacabe AG, Laplanche JL, Ryder S, Baron T (2004). Distinct molecular phenotypes in bovine prion diseases. EMBO Rep.

[42] Dudas S, Yang J, Graham C, Czub M, McAllister TA, Coulthart MB, Czub S (2010) 5Molecular, biochemical and genetic characteristics of BSE in Canada. PLoS One 5:e10638 10.1371/journal.pone.0010638 PMC2871047 20498835

[43] Espinosa JC, Andréoletti O, Castilla J, Herva ME, Morales M, Alamillo E, San-Segundo FD, Lacroux C, Lugan S, Salguero FJ, Langeveld J, Torres JM (2007). Sheep-passaged bovine spongiform encephalopathy agent exhibits altered pathobiological properties in bovine-PrP transgenic mice. J Virol.

